# The gut–microbiota–brain changes across the liver disease spectrum

**DOI:** 10.3389/fncel.2022.994404

**Published:** 2022-09-07

**Authors:** Sara G. Higarza, Silvia Arboleya, Jorge L. Arias, Miguel Gueimonde, Natalia Arias

**Affiliations:** ^1^Laboratory of Neuroscience, Department of Psychology, University of Oviedo, Oviedo, Asturias, Spain; ^2^Institute of Neurosciences of the Principality of Asturias (INEUROPA), Oviedo, Asturias, Spain; ^3^Department of Microbiology and Biochemistry of Dairy Products, Institute of Dairy Products of the Principality of Asturias (IPLA-CSIC), Villaviciosa, Asturias, Spain; ^4^Health Research Institute of the Principality of Asturias (ISPA), Oviedo, Asturias, Spain; ^5^Department of Psychology, Faculty of Life and Natural Sciences, BRABE Group, Nebrija University, Madrid, Spain

**Keywords:** liver disease, gut microbiota, object recognition, spatial working memory, cytochrome c oxidase, non-alcoholic fatty liver disease

## Abstract

Gut microbiota dysbiosis plays a significant role in the progression of liver disease, and no effective drugs are available for the full spectrum. In this study, we aimed to explore the dynamic changes of gut microbiota along the liver disease spectrum, together with the changes in cognition and brain metabolism. Sprague–Dawley rats were divided into four groups reflecting different stages of liver disease: control diet (NC); high-fat, high-cholesterol diet (HFHC), emulating non-alcoholic steatohepatitis; control diet + thioacetamide (NC + TAA), simulating acute liver failure; and high-fat, high-cholesterol diet + thioacetamide (HFHC + TAA) to assess the effect of the superimposed damages. The diet was administered for 14 weeks and the thioacetamide was administrated (100 mg/kg day) intraperitoneally over 3 days. Our results showed changes in plasma biochemistry and liver damage across the spectrum. Differences in gut microbiota at the compositional level were found among the experimental groups. Members of the *Enterobacteriaceae* family were most abundant in HFHC and HFHC + TAA groups, and *Akkermansiaceae* in the NC + TAA group, albeit lactobacilli genus being dominant in the NC group. Moreover, harm to the liver affected the diversity and bacterial community structure, with a loss of rare species. Indeed, the superimposed damage group (HFHC + TAA) suffered a loss of both rare and abundant species. Behavioral evaluation has shown that HFHC, NC + TAA, and HFHC + TAA displayed a worsened execution when discriminating the new object. Also, NC + TAA and HFHC + TAA were not capable of recognizing the changes in place of the object. Furthermore, working memory was affected in HFHC and HFHC + TAA groups, whereas the NC + TAA group displayed a significant delay in the acquisition. Brain oxidative metabolism changes were observed in the prefrontal, retrosplenial, and perirhinal cortices, as well as the amygdala and mammillary bodies. Besides, groups administered with thioacetamide presented an increased oxidative metabolic activity in the adrenal glands. These results highlight the importance of cross-comparison along the liver spectrum to understand the different gut–microbiota–brain changes. Furthermore, our data point out specific gut microbiota targets to design more effective treatments, though the liver–gut–brain axis focused on specific stages of liver disease.

## Introduction

Liver disease is nowadays one of the major health concerns of western societies. Non-alcoholic fatty liver disease (NAFLD), primarily defined by hepatic steatosis, is known to affect 25% of the population worldwide (Younossi et al., 2016). This disease can also progress to other pathological stages, such as non-alcoholic steatohepatitis (NASH), which is characterized by the presence of fibrosis (Chu et al., 2019) and is shown to be present in around 60% of the patients with NAFLD (Younossi et al., 2016). An advanced fibrosis status can lead to the development of cirrhosis (Traussnigg et al., 2015), which would be present in 0.6% of patients with NAFLD/NASH within 3 years, besides, hepatocellular carcinoma (Alexander et al., 2019).

Given the continuing nature of liver damage, different models must be used to address the distinct stages of the pathology. NAFLD and NASH spectra are usually derived in humans from the consumption of high-caloric diets (Ullah et al., 2019), which shows that the administration of high-fat, high-cholesterol (HFHC) diets in animal models leads successfully to the emulation of steatosis and other typical features of the disease (Higarza et al., 2019). The development of aggressive liver damage has been often addressed through the administration of hepatotoxic substances, such as thioacetamide (TAA) (Méndez et al., 2008a; Arias et al., 2013), which induces liver macronodular cirrhosis (Li et al., 2002). In addition, the one-off administration of TAA is able to provoke acute liver failure, as it has been revealed that the injection of 300 mg/kg of the animal led to necrosis and apoptosis in the liver (Mladenović et al., 2012).

Under these circumstances, it has been recently shown by randomized studies that a nutritional intervention aimed at ensuring sufficient energy intake significantly improves the survival of cirrhotic patients (Yamada et al., 2013). Those results were supported by da Silva et al. (2021) who demonstrated that a high-sucrose diet attenuates the liver injury and the catabolism condition of rats with cirrhosis induced by thioacetamide, suggesting that dietary changes could be a therapeutic option for cirrhotic patients. On the contrary, Romualdo et al. (2020) have proved that dietary interventions such as a high-fat diet may also be associated with repetitive chemical insults, such as TAA, which resemble the NASH-attributable hepatocarcinoma cases. This pathogenesis follows the “two-hit theory” where triglyceride accumulation leads to fatty liver, considered the “first hit.” Subsequently, lipotoxicity triggers an inflammatory response, the “second hit,” gradually allowing the emergence of liver fibrosis/cirrhosis, a favorable background for hepatocarcinoma (Margini and Dufour, 2016).

Being able to model liver disease in animal models has allowed the study of its systemic implications, including the associated cognitive impairment. In this way, the administration of HFHC diets has been linked with diverse behavioral deficits (Higarza et al., 2019), and was also suggested to be present in NAFLD human patients (Filipović et al., 2018), whereas TAA can induce hepatic encephalopathy features (Méndez et al., 2008c), known to be displayed by patients with cirrhosis (Butterworth, 2019). Furthermore, liver function has been defined to be in close relation to gut microbiota (Visschers et al., 2013), but as far as we are concerned, no studies regarding the administration of TAA have studied its potential influence on the gut microbiota composition. Moreover, it is known that HFHC is associated with gut microbiota alterations and that these alterations could also mediate associated behavioral impairment through the liver–gut–brain axis (Higarza et al., 2019). Several studies have suggested that vagal branches innervate the entire gastrointestinal tract, liver, and portal vein among others. In addition, mechanical and chemical stimuli applied to the gastrointestinal tract could directly activate free nerve endings through specific receptors and/or mechanosensitive ion channels. These results suggested that the vagal afferent system also contributes to detecting immune-related events in the periphery and generating appropriate autonomic, endocrine, and behavioral responses *via* central reflex pathways (Berthoud and Neuhuber, 2000). This gut–liver–brain interaction has been suggested to mediate hepatic encephalopathy (Patel et al., 2015; Ahluwalia et al., 2016; Mancini et al., 2018), but there are not enough animal studies that allow us to identify the key changes in microbiota that could improve cognition under these circumstances to design potential treatments.

It is well-known that the introduction of diets such as the HFHC triggered a rapid and transient decrease in bacterial diversity. Indeed, our group (Higarza et al., 2019) has previously shown that 14 weeks under HFHC induced intestinal microbiota dysbiosis in which levels of total bacteria, as well as β- and α-diversity were decreased. We found reduced levels of Firmicutes, especially members of the *Lactobacillaceae* family, but increased levels of Bacteroidetes and Proteobacteria, which were accompanied by lower levels of the main short-chain fatty acids (SCFAs) such as acetate, propionate, and butyrate, whose production has been linked to the microorganisms altered by the diet. In this line, other authors (Minaya et al., 2020) have shown that higher energy diets led to a rapid increase in members of the family *Erysipelotrichaceae* (Firmicutes) and also depleted members of the families S24-7 (Bacteroidetes) and *Verrucomicrobiaceae* (Verrucomicrobia) during the first 4 weeks. Those results revealed the timeline progression of the development of microbiota dysbiosis induced by changes in diet consumption.

It has been reported that ceramides and other toxic lipids generated by the liver in the context of NASH pathology could mediate the adverse effects on the brain due to their capacity to cross the blood–brain barrier and, once inside the brain, cause neuroinflammation, oxidative stress, metabolic impairment, and neurotransmitter deficits (de la Monte et al., 2009). Moreover, several studies have shown that SCFAs are also used as a mitochondrial energy source in both humans and rodents (Egorin et al., 1999; Schönfeld and Wojtczak, 2016; Boets et al., 2017). Indeed, relevant levels of acetate and propionate have been reported to directly influence the brain (Perry et al., 2016; Hoyles et al., 2018). At the same time, endotoxins produced by the resident gut microbiome damage vagal afferents, which in turn trigger microglia activation in the brain (Minaya et al., 2020). These findings may suggest that gut dysbiosis and gut-derived microbiota metabolites, along with other components, may be generating a neurotoxic injury that could be reflected not only in behavioral changes but also in metabolic and functional brain regional deficits.

However, more studies are warranted to fully understand the dynamic changes of the gut microbiota and cognition in the progression of liver disease. This study aimed to investigate the differences in microbiota, brain, and systemic metabolism linked to mitochondrial dysfunction and their correlation to cognitive deficits across the spectrum of liver failure. The systemic results along the liver–gut–brain axis shed light on the opportunity to manipulate the gut microbiota and its related metabolites as effective strategies in preventing brain-associated liver disease dysfunction.

Henceforth, this study aims to assess the functioning of the liver-gut-brain axis across liver diseases. For this purpose, we use four different animal models reflecting different stages of liver damage: NC (control diet), HFHC emulating NASH, NC + TAA simulating acute liver failure, and HFHC + TAA to assess the effect of the superimposed damages. These models are evaluated at different levels including hepatic histology and plasma biochemistry, fecal microbiota, behavior, and brain and adrenal oxidative metabolism. This study is a pioneer in highlighting the dynamic changes of the gut microbiota and cognition in the progression of liver disease.

## Materials and methods

### Procurement of experimental models

**FIGURE 1:**
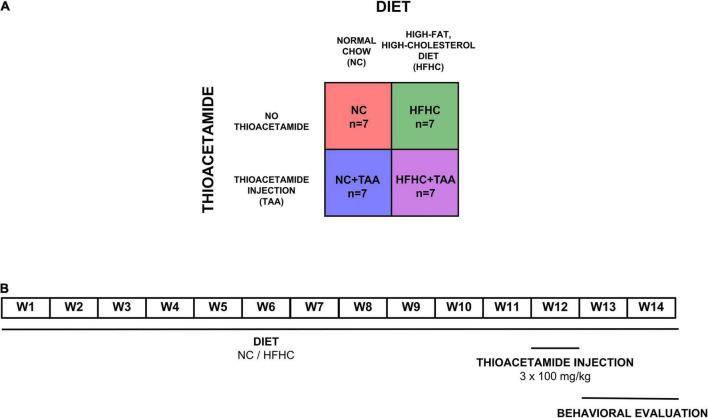
Experimental design. **(A)** Experimental groups. Four groups (*n* = 7 per experimental group) of male Sprague–Dawley rats were used. The NC group was dispensed a normal chow, the HFHC group was fed with a high-fat, high-cholesterol diet, the NC + TAA group received normal chow and thioacetamide (TAA) administration, and the HFHC + TAA group received both high-fat high-cholesterol diet and TAA. **(B)** Timeline of the experiment. Each group was administrated their respective diets for 14 weeks. On week 12, NC + TAA and HFHC + TAA received daily 3 injections of thioacetamide at a concentration of 100 mg/kg, making a total amount of 300 mg/kg. The behavioral evaluation started on week 13 to week 14 at the end of which the animals were sacrificed and the samples were collected.

Four groups (*n* = 7 per experimental group) of male Sprague–Dawley rats were used (260 g at the start of the experiment) (Envigo, Blackthorn, United Kingdom). The NC group was dispensed a normal chow, the HFHC group was fed with a high-fat, high-cholesterol diet, the NC + TAA group received normal chow and thioacetamide (TAA) administration, and the HFHC + TAA group received both high-fat high-cholesterol diet and TAA (Figure 1A).

Diets were administered for 14 weeks. Normal chow (Envigo, Blackthorn, United Kingdom; #2914) diet consisted of 13 kcal% from fat and no cholesterol, and the high-fat, high-cholesterol diet (Research Diets, New Brunswick, NJ, United States; #D09052204) contained 65 kcal% from fat, 2 kcal% from cholesterol, and 0.5% cholate to induce NASH, as we have previously described (Higarza et al., 2019). To induce acute hepatic damage, NC + TAA and HFHC + TAA groups received on the 12th week an intraperitoneal thioacetamide injection (100 mg/kg per day) over 3 days (300 mg/kg in total) (Sigma-Aldrich, St. Louis, MO, United States). Thioacetamide was dissolved in saline solution (Mladenović et al., 2012). Behavioral evaluation of all groups started at 13 weeks of administration of their respective diets (Figure 1B).

The procedures and manipulation of the animals used in this study were carried out according to the Directive (2010/63/EU), Royal Decree 53/2013 of the Ministry of the Presidency related to the protection of animals used for experimentation and other scientific purposes. All the animals had *ad libitum* tap water and were maintained at constant room temperature (22 ± 2°C), with a relative humidity of 65 ± 5% and an artificial light-dark cycle of 12 h (08:00–20:00/20:00–08:00 h).

### Behavioral evaluation

#### Locomotor activity assessment

Locomotor activity was assessed through the study of resistance to fatigue, which was carried out by using Rotarod 7750 for rats (Ugo Basile Biological Research Apparatus; Jones and Roberts, 1968). This apparatus consists of a motor-driven rotating rod, and the procedure followed consisted of two parts. In the first one, the animals are placed for 60 s on the apparatus with a constant speed of 2 rpm. In the second part, the rats are evaluated for 5 min in the accelerod test session, during which the rotation rate increases constantly until reaching 20 rpm. Rotation speed was reached when the falling off of the rod was recorded.

#### Cognitive evaluation

##### Object recognition test

This test was carried out in an open field (66 × 46 × 45 cm) of gray fiberglass with an open roof placed in a room with two diffuse white lights on its sides, which provided an illumination density of 10 lux approximately at the center of the open field. After each trial, the apparatus was cleaned with a 75% ethanol solution. The behavior of each animal was recorded by a video camera (Sony V88E) connected to a computer equipped with a computerized video-tracking system (EthoVision Pro, Noldus Information Technologies, Netherlands). The objects used were constructed from a combination of plastic pieces of different colors and shapes.

The animals were habituated for 2 days before the test. On the first day, the animals were placed first in groups and then individually in the open field for two trials of 6 min. On the second day, each one was placed in the field, with two equal objects in the center, for three trials of 6 min separated by 20 min. Two different tests were carried out pseudo-randomly on days 3 and 4: novel object (*what*) and novel place (*where*) recognition tests, respectively. Each test was composed of two phases. In the first one, each subject was exposed for 4 min in the open field to two copies of a new object (2 × object A), which were specific for each test and were located in the opposite corners at 10 cm from the walls. After a 50-min delay, each animal carried out the second trial, which was similar to the previous one, except that:

1. *Novel object*: a copy of the previously encountered object was changed to a novel one and put in the same place as before (object A + object B; same place).
2. *Novel place*: one of the two copies was placed in a different corner of the open field (2 × object A; different location).

Exploration of an object was defined as directing the nose toward the object at less than 2 cm and exploring it (i.e., sniffing and or interacting with the object). For each subject, the time spent exploring the objects in both trials was measured. The discrimination of the objects was assessed through the discrimination index (d1), the difference in time spent exploring the two objects in the second phase (novel minus familiar), and the discrimination ratio (d2), the difference in time spent exploring the two objects in the second phase divided by the total time exploring both (Ennaceur and Meliani, 1988; Mendez et al., 2015).

##### Spatial working memory test

Spatial working memory was measured in the Morris Water Maze (MWM), a cylindrical fiberglass tank measuring 150 cm in diameter with a 40-cm high wall and virtually divided into four quadrants (Morris, 1984). The water level was 30 cm at a temperature of 22 ± 2°C. The pool contained a cylindrical platform, which was 10 cm in diameter and 28 cm high, of which 2 cm was below the surface, used by the animals to escape. This apparatus was in the center of a 16-m^2^ lit room (two halogen lamps of 4,000 lux) surrounded by panels on which several extra-maze clues were ubicated. The behavior of each subject was recorded by a video camera (Sony V88E) connected to a computer equipped with a computerized video-tracking system (EthoVision Pro, Noldus Information Technologies, The Netherlands). One day before the test, the animals were habituated to the task for three trials with a platform using different starting positions in a small square water tank (47 × 75 × 38 cm).

A spatial working memory test is a paired sample task carried out for 6 days, and each session consisted of two trials (sample and retention). Within the same session, the platform, invisible to the animals, was situated in one of the four quadrants and the animal was facing the wall of another quadrant. The trial ended once the animal found the platform or when 60 s had elapsed. If the animal had not reached the hidden platform after this time, it was placed on the platform for 15 s. During the intertrial interval, the animals were placed in a bucket for 5 s. The quadrant of the platform and the starting point of the animal changed between sessions. Latencies to reach the escape platform were recorded and used as a measure of task acquisition, and velocity was used as a measure of locomotor activity. The learning criterion was considered when the animals spent significantly less time in the retention trial than in the sample trial (Higarza et al., 2019).

### Sacrifice

On week 14 of the administration of their respective diets, 90 min after the last trial of the spatial working memory task, the animals were decapitated. Blood was collected and centrifuged to collect plasma, which was frozen in *N*-methylbutane (Sigma-Aldrich, St. Louis, MO, United States) and stored at −80°C. Organs were removed and weighed. Livers were fixed with a 10% formaldehyde solution (Fisher Scientific, Hampton, VA, United States) and embedded in paraffin (Merck, Darmstadt, Germany). Brains and adrenal glands were rapidly frozen in *N*-methylbutane (Sigma-Aldrich, St. Louis, MO, United States) and stored at −40°C for the posterior study of metabolic activity. Fresh fecal samples were gathered from each animal and stored at −80°C for the latter gut microbiota study.

### Liver histological examination

Livers were fixed with a 10% formaldehyde solution and embedded in paraffin. Histology microscopic evaluation was performed in 10-μm thick sections of this tissue previously deparaffinized and stained. For this purpose, Mayers hematoxylin (Sigma-Aldrich, United States) and eosin (Leica, Germany) and conversely, Weigert’s iron hematoxylin solution and picrosirius red (Sirius Red F3BA 0.1% wt/vol in saturated picric acid; Sigma-Aldrich, United States) washed in glacial acetic acid (Probus, Madrid, Spain) and water (5:1,000) were used, followed by dehydration with ethanol (VWR, Radnor, PA, United States) and mounting in Entellan (Merck, Germany).

### Plasma biochemistry determination

Plasma biochemistry was performed using a Cobas Integra II system (Roche Diagnostics, Rotkreuz, Switzerland). Plasma was biochemically assessed for the presence of ammonia (NH_3_), creatinine, bilirubin, total cholesterol, aspartate aminotransferase (AST), and alanine aminotransferase (ALT).

### Fecal microbiota analysis

#### Fecal pellets processing

Fecal pellets were homogenized with PBS (1:5 w/v) for 3 min at full speed in a stomacher (LabBlender 400). Cell pellet and fecal water were separated by centrifugation (10,000 rpm, 15 min) and kept at −20°C for further analyses.

#### Analysis of fecal microbiota by 16S rRNA gene profiling and quantitative PCR

Bacterial DNA was isolated by using the QIAamp DNA stool kit (Qiagen, GmbH, Germany) following the manufacturer’s instructions. The hypervariable V3 region of the 16S rRNA gene was amplified using primers and conditions previously described (Milani et al., 2013), and the products were sequenced on an Illumina MiSeq platform following previously reported protocols (Milani et al., 2013). The QIIME 2 suite of tools was used for individual reads processing, including filtering, trimming, denoising, chimera detection (Nogacka et al., 2017), and operational taxonomic unit grouping (Edgar, 2010). All reads were classified to the lowest taxonomical rank using a dataset from the SILVA database (Quast et al., 2013).

Absolute levels determination of different microbial groups including, *Akkermansia*, *Bifidobacterium*, *Enterobacteriaceae*, and *Lactobacillus* group, were carried out by qPCR using the following primers: (BifF: GATTCTGGCTCAGGATGAACGC, BifR: CTGATAGGACGCGACCCCAT; AkkF: CAGCACGTG AAGGTGGGGAC, AkkR: CCTTGCGGTTGGCTTCAGAT; EnterF-CATTGACGTTACCCGCAGAAGAAGC, EnterR-CTC TACGAGACTCAAGCTTGC; LactF: AGCAGTAGGGAATCT TCCA, LactR: CATGGAGTTCCACTGTCCTC) with conditions previously described (Arboleya et al., 2012).

#### Analyses of short-chain and branched-chain fatty acids in feces

Fecal water was used for the determination of the levels of short-chain fatty acids (SCFAs) and branched-chain fatty acids (BCFAs) by gas chromatography as previously described (Moris et al., 2018).

### Oxidative metabolism

Metabolic activity was assessed through the histochemistry of cytochrome c oxidase (CCO), which is based on the method developed by Gonzalez-Lima and Cada (1994), consisting of a modified version of the protocol described by Wong-Riley (1989). Brains and adrenal glands that were extracted and frozen 90 min after the last trial of the spatial working memory task were processed in 30-μm-thick coronal sections using a cryostat microtome (Leica, Wetzlar, Germany; #CM1900) and subsequently mounted on slides. To control staining variability across different baths, sets of the brain and also adrenal tissue homogenate standards of known CCO activity from rats were cut at different thicknesses (10, 30, 50, and 70 μm) and included with each batch of slides. Sections and standards were fixed with 0.5% (v/v) glutaraldehyde (Merck, Darmstadt, Germany) and 10% (w/v) sucrose (Sigma-Aldrich, St. Louis, MO, United States) in phosphate buffer (0.1 M, pH 7.6) solution. Next, the slides were rinsed three times in 10% (w/v) sucrose (Sigma-Aldrich, St. Louis, MO, United States) in phosphate buffer (0.1 M, pH 7.6) and immersed in a Tris (Sigma-Aldrich, St. Louis, MO, United States) buffer solution (0.05 M, pH 7.6) containing 0.5% (v/v) dimethylsulfoxide (Fisher Scientific, Hampton, VA, United States), 10% (w/v) sucrose (Sigma-Aldrich, St. Louis, MO, United States), and 275 mg/L cobalt chloride (Sigma- Aldrich, St. Louis, MO, United States). Once the slides were rinsed in a phosphate buffer (0.1 M, pH 7.6), they were incubated in the dark at 37°C in a PBS solution (0.1 M, pH 7.6) containing 0.0075% (w/v) cytochrome c (Sigma-Aldrich, St. Louis, MO, United States), 0.002% (w/v) catalase (Alfa Aesar, Haverhill, NH, United States), 5% (w/v) sucrose (Sigma-Aldrich, St. Louis, MO, United States), 0.25% (v/v) dimethylsulfoxide (Fisher Scientific, Hampton, VA, United States), and 0.05% (w/v) diaminobenzidine tetrahydrochloride (Sigma-Aldrich, St. Louis, MO, United States) for 1 h. The reaction was stopped by fixing the tissue in 4% (v/v) buffered formalin (Sigma-Aldrich, St. Louis, MO, United States) with 10% (w/v) sucrose (Sigma-Aldrich, St. Louis, MO, United States). Finally, the slides were dehydrated, cleared with xylene, and coverslipped with Entellan (Merck, Darmstadt, Germany).

The CCO histochemical staining intensity was quantified by densitometric analysis using the computer-assisted image analysis workstation MCID (Interfocus Imaging, Linton, United Kingdom), which consists of a high precision illuminator, a digital camera, and a computer with specific image analysis software. The mean optical density of each region was measured by taking 12 readings which were then averaged to obtain one mean per region for each animal. These optical density values were then converted to CCO activity units (μmol of cytochrome c oxidized/min/g tissue wet weight), determined by the enzymatic activity of the standards measured spectrophotometrically.

Neuronal metabolic activity was studied in selected brain regions anatomically defined according to Paxinos and Watson’s (2004). The regions of interest and their distance from bregma were as follows: the prefrontal cortex [+3.24 mm; prelimbic (PrL), infralimbic (IL), and cingulate (Cg) cortices], the amygdala [-2.28 mm; central (CeA), lateral (LaA), and basolateral (BLA)], the dorsal hippocampus [-3.00 mm; dentate gyrus (DG), CA1 and CA3 areas], the retrosplenial cortices [-3.84 mm; granular (RSG) and dysgranular (RSD)], the perirhinal (PRh) and entorhinal (Ent) cortices (-4.20 mm), and the mammillary bodies [-4.44 mm; medial nucleus (mMM), the lateral part of the medial nucleus (lMM), the lateral mammillary nucleus (LM), and the supramammillary nucleus (SuM)]. Moreover, oxidative metabolism was studied in the cortex of the adrenal glands, specifically in the zona glomerulosa, zona fasciculata, and zona glomerularis.

### Statistical analyses

Data derived from gut microbiota were analyzed using IBM SPSS Statistics (IBM Corp., Armonk NY, United States), R, and MicrobiomeAnalyst software. Total sum normalization and cumulative-sum scaling to account for the non-normal distribution of taxonomic count data for diversity and abundance analyses were used respectively (Chong et al., 2020). Beta diversity profiling was estimated based on principal coordinates analysis with Bray-Curtis metrics, and permutational Manova (Permanova) was calculated. Alpha diversity was analyzed based on different indexes (Chao1 and Shannon) and their normal distribution. Further differential abundance analyses were performed through DeSeq2, and a linear discriminant analysis effect size (LefSe; with an effect-size threshold of 2.0) for searching for biomarkers was carried out. Data from qPCR and SCFAs were compared through the Kruskal–Wallis or ANOVA tests. *Post-hoc* multiple comparisons analyses were carried out, when allowed, using Dunn’s or Tukey’s method, respectively. Data were considered statistically significant at *p* < 0.05.

Data related to physiology and neurobiology were analyzed in the Sigmaplot 12.0 program (Systat, United States) and expressed as means ± SEM. A two-way repeated-measures ANOVA (group x week) was used to compare weight gain across weeks. A one-way ANOVA was used to compare the final body weight, the relative organ weights, the biochemistry values in plasma, and the cytochrome c oxidase values between groups. Maximum speed spent on the Rotarod was compared through the Kruskal–Wallis one-way analysis of variance. In the object recognition test, the exploration displayed in both trials (e1 and e2) was studied through a paired *t*-test, the discrimination index (d1) and discrimination ratio (d2) were compared with zero (chance performance indicating equal exploration of the two objects or locations) through a *t*-test for independent samples, and d1 and d2 values were compared between groups through a one-way ANOVA. In the spatial working memory test, sample and retention trials latencies were compared within each group through a paired *t*-test. Latencies presented within sample and retention trials and velocity to perform the task along the days were compared through a two-way repeated-measures ANOVA (group × day). *Post-hoc* multiple comparisons analyses were carried out, when allowed, using Tukey’s method. The Kruskal–Wallis one-way analysis of variance on ranks and the Mann–Whitney *U* test for independent samples were applied when normality or equal group variances failed. The results were considered statistically significant if the *p-*value was < 0.05.

## Results

### Body weight and organ relative weight

**FIGURE 2:**
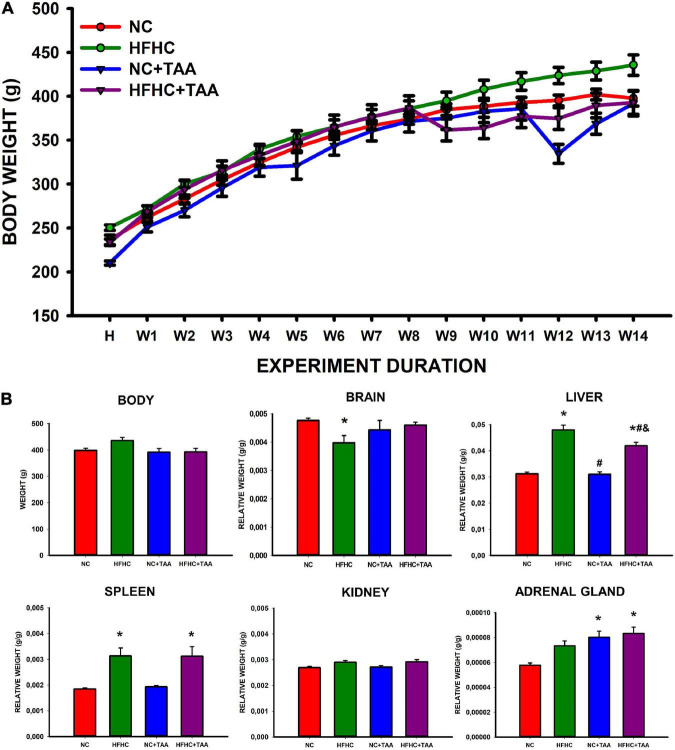
Body and organs weight. **(A)** Body weight across experimental weeks. The Scatter plot (mean ± SEM) represents body weight, which was compared through a two-way repeated-measures ANOVA (group X week). H, habituation week; W, week. No differences were found between the groups, but the weight increased significantly across the weeks (*p* < 0.001). No statistically significant differences were neither observed in the final body weight when compared through a one-way ANOVA. **(B)** Body and organs relative weight. Bars charts (mean ± SEM) represent body weight (g) as well as the brain, liver, spleen, kidney, and adrenal gland relative weight (g/g) (organ weight/body weight) compared through a one-way ANOVA followed by Tukey’s test (*comparison with NC, ^#^comparison with HFHC, ^&^comparison with NC + TAA; *^#&^*p* < 0.05).

The study of the body weight between groups and along the weeks revealed an interaction between these two factors (group × week; *F*_42_, _336_ = 7.110, *p* < 0.001). Thus the effect of the group depends on the week in which is studied. There was no significant difference in the body weight between groups (*F*_3_, _24_ = 2.373, *p* = 0.095), but it did increase across the weeks as expected (*F*_14_, _336_ = 616.899, *p* < 0.001) (Figure 2A). When studying the final body weights, no statistically significant differences were observed (*F*_3_, _24_ = 2.967, *p* = 0.052) (Figure 2B).

Regarding the organ relative weight (organ weight/body weight), some differences were found (Figure 2B). HFHC showed a reduced brain relative weight compared with NC (*H*_3_ = 9.752, *p* = 0.021). Liver relative weight was significantly different between groups (*F*_3_, _24_ = 40.872, *p* < 0.001), as HFHC and HFHC + TAA displayed a higher value than NC and NC + TAA and also did HFHC when compared with HFHC + TAA. Changes in the splenic relative weight were also observed (*H*_3_ = 12.686, *p* = 0.005) as HFHC and HFHC + TAA showed an increased value with respect to the NC group. No statistically significant differences between groups were found in the relative weight of the kidneys (*F*_3_, _24_ = 2.882, *p* = 0.057); however, NC + TAA and HFHC + TAA presented an increased value of the weight of adrenal glands in comparison with the NC group (*F*_3_, _24_ = 7.774, *p* < 0.001).

**FIGURE 3:**
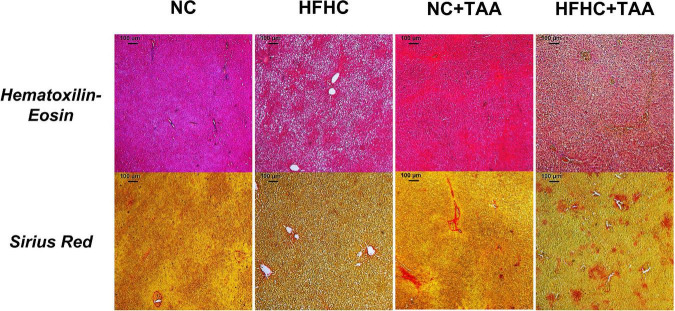
Microscopic examination (5×) of each experimental group. Liver samples were stained with Hematoxylin–Eosin and Sirius Red. HFHC and HFHC + TAA group presented steatosis; moreover, NC + TAA and HFHC + TAA showed progressive fibrosis accumulation and hepatic damage.

Finally, the liver microscopic examination revealed that the HFHC group exhibited steatosis, and NC + TAA and HFHC + TAA displayed progressive fibrosis accumulation and hepatic damage (Figure 3).

### Plasma biochemistry determination

**FIGURE 4:**
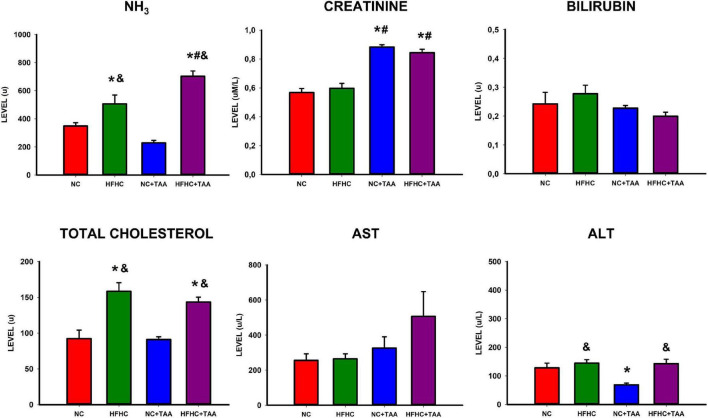
Biochemical assessment in plasma. Bars charts (mean ± SEM) represent levels of ammonia (NH_3_), creatinine, bilirubin, total cholesterol, aspartate aminotransferase (AST), and alanine aminotransferase (ALT) (u) compared through a one-way ANOVA followed by Tukey’s test (*comparison with NC, ^#^comparison with HFHC, ^&^comparison with NC + TAA; *^#&^*p* < 0.05).

The study of plasma biochemistry revealed some differences between groups (Figure 4). Significant differences were found in ammonia (*F*_3_, _26_ = 24.519, *p* < 0.001) between experimental groups with higher levels in the HFHC group when compared to NC (*p* ≤ 0.05) and NC + TAA (*p* < 0.001). Besides, increased ammonia values were found in the HFHC + TAA group when compared to NC (*p* < 0.001), HFHC (*p* = 0.011), and NC + TAA (*p* < 0.001).

Moreover, increased creatinine values were found in thioacetamide groups (*F*_3_, _25_ = 35.893, *p* < 0.001) with differences between NC + TAA vs. NC (*p* < 0.001) and HFHC (*p* < 0.001), and differences were found between HFHC + TAA vs. NC (*p* < 0.001) and HFHC (*p* < 0.001) as well.

Furthermore, total cholesterol was increased in the HFHC groups (*F*_3_, _24_ = 13.839, *p* < 0.001) with differences between HFHC vs. NC (*p* < 0.001) and NC + TAA (*p* < 0.001), as well as differences between HFHC + TAA vs. NC (*p* = 0.006) and NC + TAA (*p* = 0.003). This increase in cholesterol was accompanied by an increase in ALT values (*F*_3_, _25_ = 6.706, *p* = 0.002) between HFHC and NC + TAA (*p* = 0.003), NC + TAA and NC (*p* = 0.032), and between HFHC + TAA and NC + TAA (*p* = 0.005).

No statistically significant differences were observed between groups in bilirubin (*F*_3_, _25_ = 1.704, *p* = 0.195) or AST (*F*_3_, _25_ = 1.965, *p* = 0.149) values.

### Fecal microbiota analysis

#### Diversity assessment

**FIGURE 5:**
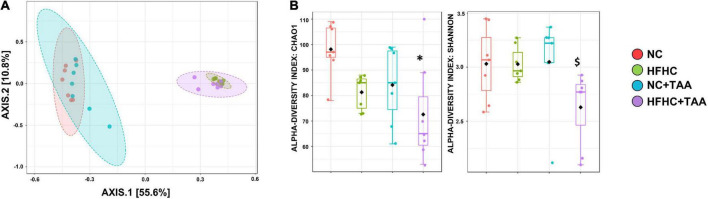
Gut microbiota diversity. **(A)** Principal coordinate (PCoA) analysis based on Bray–Curtis distances. The PERMANOVA test showed a clear separation (*p* < 0.001) of the microbiotas among the groups studied. **(B)** Box-and-whiskers (median and IRQ range) represents the comparison of alpha-diversity of gut microbiota using the Chao1 and Shannon indexes. The one-way ANOVA (*p* < 0.016) followed Tukey’s test and the Kruskal–Wallis (*p* < 0.037) followed by Dunn’s test, respectively, showed differences among groups studied (*comparison with NC group; ^$^comparison with all the groups). *^$^*p* < 0.05.

The 16S sequencing revealed substantial differences in the diversity and composition among the groups. Principal coordinate analysis based on the Bray-Curtis indexes, measuring the between-group differences in diversity, showed a clear separation (*p* < 0.001) of the microbiotas in two clusters, mainly based on the type of diet. However, the pairwise comparisons showed differences between groups with the same diet. The lowest significant effect was observed between the NC and NC + TAA groups with a lower *R*^2^ value (0.177) and *p* < 0.016, while the pairwise differences between the rest of the groups were higher (*p* < 0.002) (Figure 5A). Alpha diversity significantly changed among groups based on several measures, including Chao 1 (*p* < 0.05) and Shannon (*p* < 0.05) estimators. Chao 1 was the highest in the NC group, and a significantly lower richness (*p* = 0.010) was observed in the HFHC + TAA group with respect to the NC group. A significantly lower evenness (*p* < 0.05) was also found in the HFHC + TAA group with regard to the other three experimental groups (Figure 5B).

#### Microbiota composition

**FIGURE 6:**
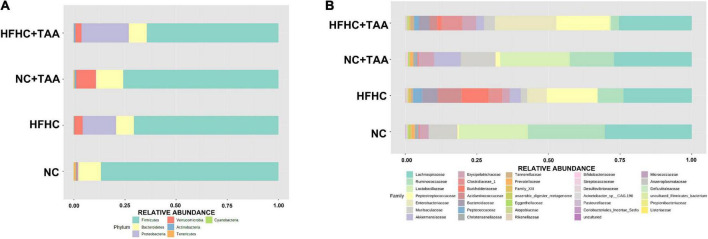
Gut microbiota composition. Average relative abundances of gut microbiota **(A)** at the phylum level and **(B)** at the family level from the NC, HFHC, NC + TAA, and HFHC + TAA groups.

Firmicutes were the main phylum in the fecal microbiota of all the groups followed by Bacteroidetes in the NC and NC + TAA groups (without reaching significant differences among groups) and Proteobacteria (*p* < 0.05) in HFHC and HFHC + TAA. Relative proportions of Verrucomicrobia were significantly lower (*p* < 0.001) in the NC group with respect to the other three, and the NC + TAA group showed higher levels than HFHC + TAA (*p* < 0.05) (Figure 6A). Analysis of the data at lower taxonomical levels confirmed these observations. *Lachnospiraceae*, belonging to Firmicutes, was the family dominant in all the groups. The NC group showed high relative abundances of *Lactobacillaceae* and *Ruminococacceae*, as well as NC + TAA. The HFHC group displayed high abundances of *Peptostreptococcaceae* and *Burkholderiaceae*, and the HFHC + TAA group showed increased relative abundances of *Enterobacteriaceae* and *Peptostreptococcaceae* as the main families (Figure 6B).

**FIGURE 7:**
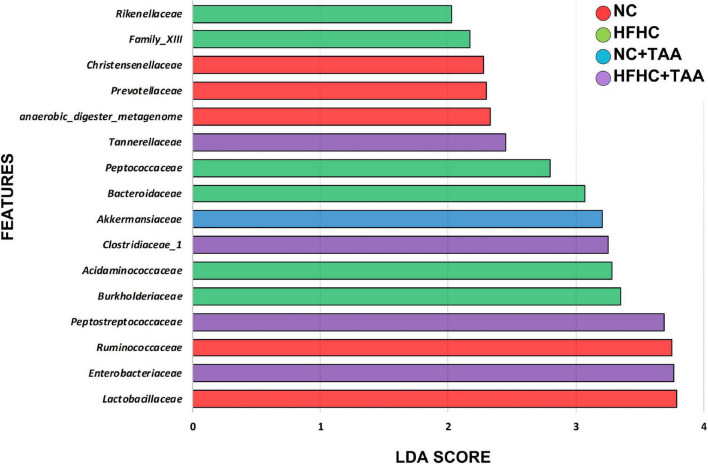
Gut microbiota differences. Results of LEfSe analysis (LDA scores > 2 and significance of *p* < 0.05 as determined by Wilcoxon’s signed-rank test) show taxa are significantly different among experimental groups. Red indicates discriminant families in the NC group; green indicates discriminant families in the HFHC group; blue indicates discriminant families in the NC + TAA group; and purple indicates in the HFHC + TAA group.

To explore how the different liver injury levels could affect the different gut microbiota taxa, a linear discriminant analysis effect size (LEfSe) method was applied at the family level to investigate the phylotypes most likely to explain differences in abundance among the groups. When we compared the four animal groups, namely NC, HFHC, NC + TAA, and HFHC + TAA, there were six bacterial families that discriminated the HFHC group from the others, with *Burkholderiaceae*, a Proteobacteria family, having the greatest discriminatory power, followed by *Acidaminococcaceae* (Firmicutes members). The NC + TAA group was discriminated by higher *Akkermansiaceae*, and for the superimposed group, HFHC + TAA, several discriminative bacteria were identified, with *Enterobacteriaceae* having the greatest discriminatory power, followed by *Peptostreptococcaceae* and *Clostridiaceae* 1 among others. *Lactobacillaceae* and *Ruminococacceae* were associated with the NC group (Figure 7).

**FIGURE 8:**
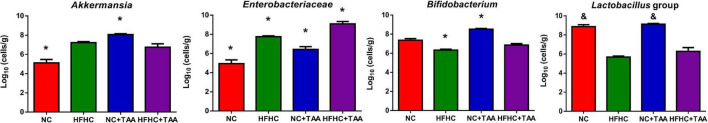
Bacterial concentration. Bars charts (mean ± SEM) represent the concentration of *Akkermansia* genus, *Enterobacteriaceae* family, *Bifidobacterium* genus, and *Lactobacillus* group analyzed by qPCR (log_10_ cells/g feces). Comparisons through Kruskal–Wallis (*Akkermansia, p* < 0.000; *Lactobacillus*, *p* < 0.000) followed by Dunn’s test and the one-way ANOVA (*Enterobacteriaceae, p* < 0.000; *Bifidobacterium*, *p* < 0.000) followed Tukey’s test, showed differences among groups studied (*p < 0.05, comparison with all the groups; ^&^*p* < 0.05, comparison with HFHC and HFHC + TAA groups).

Since feature selection with LEfSe depends on the groups included in the analysis and since we wanted to unveil in deep which bacteria could discriminate each TAA group from its control, we conducted LEfSe pairwise comparisons. When we compared the NC + TAA group with the NC group, *Akkermansia* continued to be the discriminant bacterial taxon in the acute illness by thioacetamide (Supplementary Figure 1). Moreover, qPCR data confirmed a higher concentration of *Akkermansia* in the NC + TAA group (Figure 8). However, when HFHC and HFHC + TAA groups are compared, the genera which can distinguish between them are *Parasuttella* and *Phascolarctobacterium* in the HFHC group, and *Eubacterium fissicatena* group and *Lachnoclostridium* in the HFHC + TAA group. Finally, the comparison between the two cirrhosis groups showed *Lactobacillus* and *Lachnospiraceae* NK4A136 group with the highest discriminant power in the NC + TAA group, and *Escherichia/Shigella* and *Romboutsia* as the most powerful features discriminant in HFHC + TAA rats. Moreover, qPCR data also confirmed higher lactobacilli concentration in the NC + TAA group (Supplementary Figure 1 and Figure 8).

*Enterobacteriaceae*, a Proteobacteria family comprising many pathogenic microorganisms, is strengthened by the conditions present in the liver disease context. Its concentration is significantly increased (*p* < 0.000) in the HFHC group when compared to the control group, but it is also increased in the acute diseases, NC + TAA (*p* < 0.001) and HFHC + TAA (*p* < 0.01), groups. *Bifidobacterium* did not appear as a discriminant taxon in the Lefse analysis for any disease stage; however, qPCR results showed an interesting shift. The concentration of this genus was significantly lower (*p* < 0.05) in HFHC and higher (*p* < 0.000) in NC + TAA groups with respect to the other groups (Figure 8).

#### Microbiota metabolism

**FIGURE 9:**
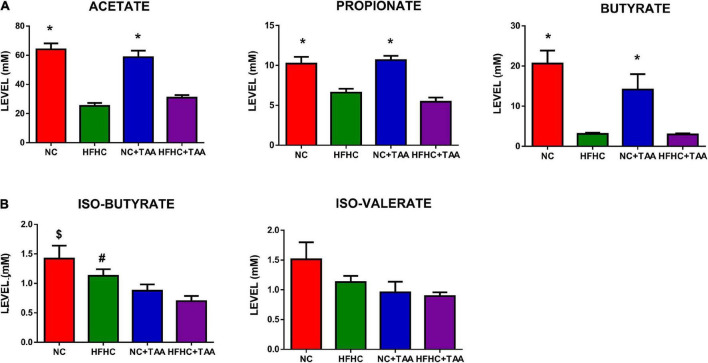
SCFA and BCFAs levels. Bars charts (mean ± SEM) represent the **(A)** SCFAs and **(B)** BCFAs levels (mM) compared through the Kruskal–Wallis test followed by Dunn’s analysis (*comparison with HFHC and HFHC + TAA groups; ^$^comparison with NC + TAA and HFHC + TAA groups; ^#^comparison with HFHC + TAA group; *^#$^*p* < 0.05).

With the shifts shown in the microbiota composition among the groups, it was not surprising to observe changes in the SCFAs and BCFAs levels in the gut lumen of those experimental groups. Acetate was the SCFAs with higher concentration, followed by butyrate and propionate in NC and NC + TAA groups, but HFHC and HFHC + TAA groups showed higher levels of propionate than butyrate (Figure 9A). However, when minority acids were analyzed (BCFAs), a gradual decrement in the concentration of iso-butyrate and iso-valerate was observed with liver illness diseases (Figure 9B). The NC + TAA group showed lower (*p* < 0.05) iso-butyrate concentration than the NC group, and HFHC + TAA showed lower values than NC (*p* < 0.01) and HFHC (*p* < 0.05) groups. A similar trend, but without reaching statistical significance, was observed for iso-valerate (Figure 9B).

### Behavioral evaluation

#### Locomotor activity assessment

**FIGURE 10:**
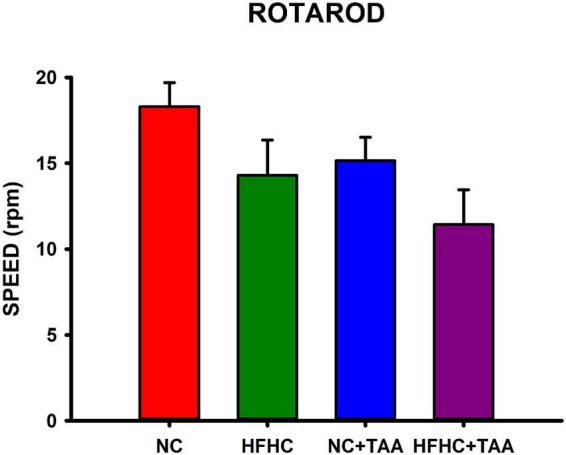
Locomotor function evaluation measured in Rotarod-accelerod test. Bars charts (mean ± SEM) represent the maximum speed (rpm) that the animals spent on the rod, which was compared through the Kruskal–Wallis test. There were no statistically significant differences between groups.

Locomotor activity was evaluated in the Rotarod-accelerated test. Results showed that there were no statistically significant differences between groups in the maximum speed that the animals were able to stay in the rod (*H*_3_ = 7.655, *p* = 0.054), thus they did not display altered locomotor activity in this test (Figure 10).

#### Cognitive evaluation

##### Object recognition test

###### Novel object

**FIGURE 11:**
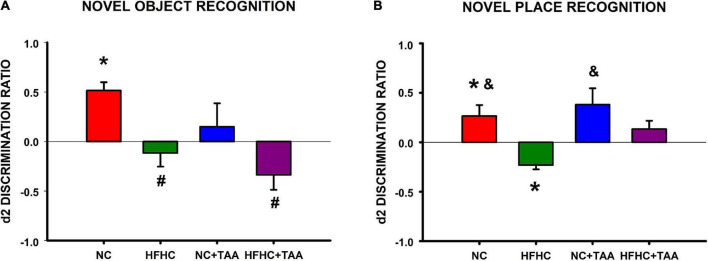
Object recognition test. **(A)** Novel object recognition test. Bars charts (mean ± SEM) represent the discrimination ratio (d2) between the new object and the one previously observed. This value was analyzed through a *t*-test for independent samples (*comparison with zero) and a one-way ANOVA followed by Tukey’s test (^#^comparison with NC d2 value; ^&^comparison with HFHC d2 value; *^#&^*p* < 0.05). NC was able to discriminate against the new object, whereas HFHC, NC + TAA, and HFHC + TAA were not able to discriminate against it. HFHC and HFHC + TAA displayed a lower d2 ratio than NC; NC + TAA did not show differences from the NC group but an impairment in novel object recognition was found. **(B)** Novel place recognition test. Bars charts (mean ± SEM) represent the discrimination ratio (d2) between the new location and the one previously used. This value was analyzed through a *t*-test for independent samples (* comparison with zero) and a one-way ANOVA followed by Tukey’s test (# comparison with NC d2 value; ^&^comparison with HFHC d2 value; *^#&^*p* < 0.05). NC showed a preference for the new location, whereas NC + TAA and HFHC + TAA were not able to discriminate the new place of the object. Moreover, HFHC spent more time exploring the object in the previous location and it displayed a lower d2 ratio than NC and NC + TAA.

In the novel object recognition test, NC, HFHC, and NC + TAA groups displayed a similar exploration in the two trials of the test since there were no statistically significant differences between e1 and e2 (NC: *t*_6_ = −0.566, *p* = 0.592; HFHC: *t*_6_ = 0.337, *p* = 0.748; NC + TAA: *t*_6_ = −0.526, *p* = 0.618). However, HFHC + TAA showed an increased exploration in the second trial (*t*_6_ = −2.909, *p* = 0.027). NC group was able to recognize the novel object as d1 and d2 were statistically significantly higher than zero (d1: *U* = 0.000, *n_1_* = 7, *n_2_* = 7, *p* < 0.001; d2: *U* = 0.000, *n_1_* = 7, *n_2_* = 7, *p* < 0.001). Nevertheless, this was not achieved by HFHC (d1: *U* = 21.000, *n_1_* = 7, *n_2_* = 7, *p* = 0.710; d2: *U* = 21.000, *n_1_* = 7, *n_2_* = 7, *p* = 0.710), NC + TAA (d1: *U* = 24.500, *n_1_* = 7, *n_2_* = 7, *p* = 1.000; d2: *U* = 24.500, *n_1_* = 7, *n_2_* = 7, *p* = 1.000), or HFHC + TAA (*U* = 17.500, *n_1_* = 7, *n_2_* = 7, *p* = 0.383; d2: *U* = 17.500, *n_1_* = 7, *n_2_* = 7, *p* = 0.383) whose d1 and d2 values did not differ from zero. On comparing the discrimination ratio d2 between groups, it was found that this value was significantly higher in the NC group compared with HFHC and HFHC + TAA (*F*_3_, _24_ = 5.149, *p* = 0.007) (Figure 11A). Thus, HFHC, NC + TAA, and HFHC displayed worsened novel object recognition.

###### Novel place

In the novel place recognition test, NC, HFHC, and NC + TAA groups displayed a similar exploration in the two trials of the test since there were no statistically significant differences between e1 and e2 (NC: *t*_6_ = −0.921, *p* = 0.392; HFHC: *t*_6_ = 0.727, *p* = 0.495; NC + TAA: *t*_6_ = −1.439, *p* = 0.200). However, HFHC + TAA showed an increased exploration in the second trial (*t*_6_ = −3.481, *p* = 0.013). NC group preferred the new location of the object as d1 and d2 were statistically significantly higher than zero (d1: *t*_12_ = 2.429, *p* = 0.032; d2: *t*_12_ = 2.384, *p* = 0.035), whereas HFHC interacted significantly more with the previous location as its d1 and d2 values were statistically significantly lower than zero (d1: *U* = 0.000, *n_1_* = 7, *n_2_* = 7, *p* < 0.001; d2: *t*_12_ = −5.150, *p* < 0.001). On its part, NC + TAA (d1: *U* = 14.000, *n_1_* = 7, *n_2_* = 7, *p* = 0.209; d2: *U* = 14.000, *n_1_* = 7, *n_2_* = 7, *p* = 0.209) and HFHC + TAA (d1: *t*_12_ = 1.209, *p* = 0.250; d2: *t*_12_ = 1.619, *p* = 0.131) did not prove to be aware that the object has moved from its location. On comparing the discrimination ratio d2 between groups, it was found that this value was significantly lower in the HFHC group compared with NC and NC + TAA (*F*_3_, _24_ = 5.828, *p* = 0.004) (Figure 11B).

##### Spatial working memory test

**FIGURE 12:**
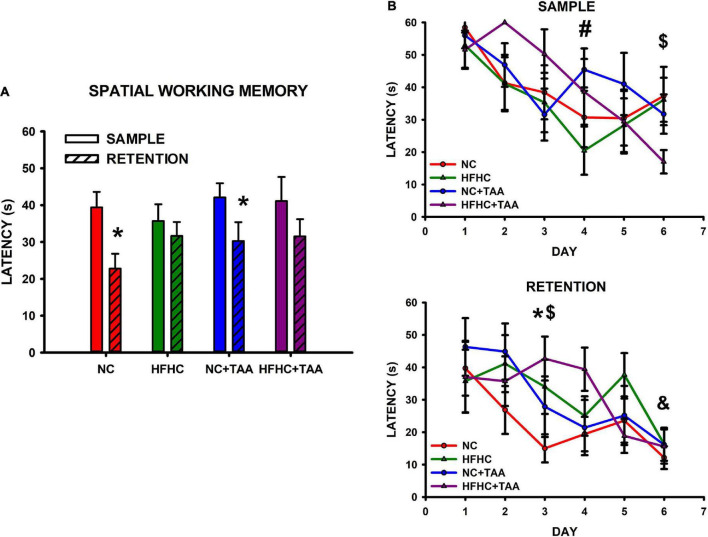
Spatial working memory test. **(A)** Bars charts (mean ± SEM) represent the average latency in sample and retention trials that was compared through paired *t*-test (* comparison with its respective sample; *p < 0.05). The NC and NC + TAA groups remembered the position of the platform in the retention trial, whereas HFHC and HFHC + TAA were not able to. **(B)** Scatter plots (mean ± SEM) represent latencies that were compared through a two-way repeated-measures ANOVA (group x day) within sample and retention. No differences were found between groups; however, a delay in the acquisition of the affected groups was detected. In the sample trials, the HFHC group displayed significant differences in latencies on day 4 compared to those on day 1 (#*p* < 0.05), whereas HFHC + TAA showed reduced latencies on day 6 when compared with those on days 1, 2, and 3 ($*p* < 0.05). In the retention trials, differences were observed in the NC + TAA group on day 6 compared with day 1 (&*p* < 0.05) and on day 3 in the NC (*p < 0.05) and HFHC + TAA ($*p* < 0.05) groups.

In the spatial working memory test, NC and NC + TAA were able to remember the position of the platform, as they showed a significant reduction in the latency in the retention in comparison with the sample trials (NC: *t*_5_ = 5.743, *p* = 0.002; NC + TAA: *t*_5_ = 3.481, *p* = 0.018), whereas HFHC and HFHC + TAA were not capable of remembering it (HFHC: *t*_5_ = 0.840, *p* = 0.439; HFHC + TAA: *t*_5_ = 2.560, *p* = 0.051) (Figure 12A). When comparison within trials took place, it was found that there were no statistically significant differences between groups in the latencies displayed neither in the sample (*F*_3_, _24_ = 0.977, *p* = 0.420) nor in the retention (*F*_3_, _24_ = 1.355, *p* = 0.280) trials (Figure 12B). However, significant differences along training days were found in sample trials (*F*_5_, _120_ = 6.319, *p* < 0.001) between days 1 and 4 in the HFHC group (*p* = 0.046), and between day 6 and the 3 first days of the training in the HFHC + TAA group (*p* < 0.04). Moreover, differences in retention (*F*_5_, _120_ = 6.208, *p* < 0.001) were found between days 1 and 6 (*p* = 0.049) in the NC + TAA group, and on day 3 between NC and HFHC + TAA groups (Figure 12B). Regarding the velocity displayed by the animals across the sessions, there were no statistically significant differences between groups (*F*_15_, _12_ = 0.959, *p* = 0.538), but this was reduced along the days (*F*_5_, _60_ = 2.691, *p* = 0.029). Thus, an impaired execution of the spatial working memory task was observed in HFHC and HFHC + TAA animals not derived from a locomotor malfunction. Moreover, the administration of TAA caused a delay in the acquisition of the task.

### Oxidative metabolism

#### Brain oxidative metabolism

The study of the CCO activity revealed some differences as HFHC + TAA displayed elevated CCO values. In this way, this group showed increased CCO values compared with the NC group in the lateral amygdala (LaA; *F*_3_, _23_ = 5.467, *p* = 0.006) and compared with NC + TAA groups in the medial mammillary nucleus (mMM; *F*_3_, _20_ = 5.285, *p* = 0.008), lateral part of the medial mamillary nucleus (lMM; *H*_3_ = 8.991, *p* = 0.029), and supramammillary nucleus (SuM; *F*_3_, _20_ = 3.236, *p* = 0.044). Besides, HFHC + TAA displayed higher CCO values than HFHC in the prelimbic cortex (PrL; *F*_3_, _23_ = 3.338, *p* = 0.037), cingulate cortex (Cg; *H*_3_ = 7.978, *p* = 0.046), lateral amygdala (LaA; *F*_3_, _23_ = 5.467, *p* = 0.006), granular retrosplenial cortex (RSG; *H*_3_ = 8.962, *p* = 0.030), dysgranular retrosplenial cortex (RSD; *F*_3_, _24_ = 3.157, *p* = 0.043), perirhinal cortex (PRh; *F*_3_, _23_ = 3.421, *p* = 0.034), and medial mammillary nucleus (mMM; *F*_3_, _20_ = 5.285, *p* = 0.008).

**FIGURE 13:**
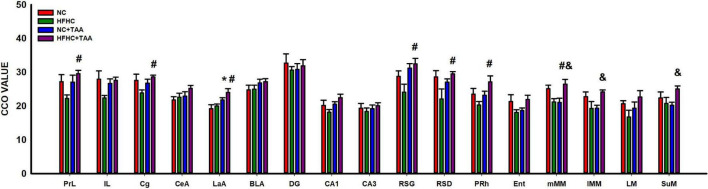
Brain oxidative metabolism. Bars charts (mean ± SEM) represent CCO values. The studied regions were: the prefrontal cortex [prelimbic (PrL), infralimbic (IL), and cingulate (Cg) cortices], the amygdala [central (CeA), lateral (LaA), and basolateral (BLA)], the dorsal hippocampus [dentate gyrus (DG), CA1 and CA3 areas, the retrosplenial (granular (RSG), and dysgranular (RSD)], the perirhinal (PRh) and entorhinal (Ent) cortices and the mammillary bodies [medial nucleus (mMM), the lateral part of the medial nucleus (lMM), the lateral mammillary nucleus (LM), and the supramammillary nucleus (SuM)]. These values were compared through a one-way ANOVA followed by Tukey’s test (*comparison with NC, ^#^comparison with HFHC, ^&^comparison with NC + TAA; *^#&^*p* < 0.05).

No statistically significant differences were observed in the CCO values between groups in the infralimbic cortex (IL; *F*_3_, _23_ = 2.441, *p* = 0.090), central amygdala (CeA; *F*_3_, _23_ = 1.789, *p* = 0.177), basolateral amygdala (BLA; *F*_3_, _23_ = 1.216, *p* = 0.326), dentate gyrus (DG; *F*_3_, _24_ = 0.254, *p* = 0.858), CA1 (*F*_3_, _24_ = 2.618, *p* = 0.074), CA3 (*F*_3_, _24_ = 0.385, *p* = 0.765), entorhinal cortex (Ent; *H*_3_ = 5.450, *p* = 0.142), and lateral mammillary nucleus (LM; *F*_3_, _19_ = 1.775, *p* = 0.186) (Figure 13).

#### Adrenal oxidative metabolism

**FIGURE 14:**
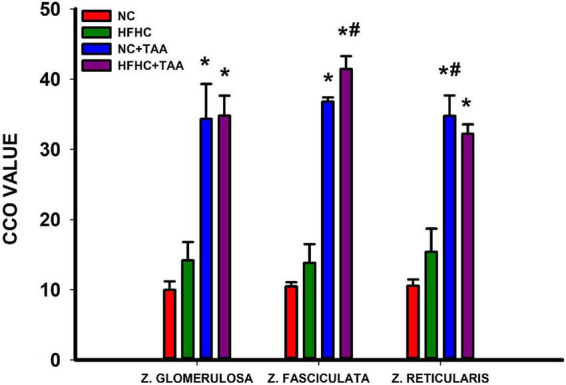
Adrenal oxidative metabolism. Bars charts (mean ± SEM) represent CCO values. The studied regions were the zona glomerulosa, the zona fasciculata, and the zona reticularis. These values were compared through a one-way ANOVA followed by Tukey’s test (*comparison with NC, ^#^comparison with HFHC; *^#^*p* < 0.05).

The CCO technique in the adrenal glands revealed some differences in the metabolic activation in the cortex of the adrenal glands. The groups administered with TAA, NC + TAA, and HFHC + TAA displayed elevated oxidative metabolism in all the areas measured in comparison with the NC group, including zona glomerulosa (*H*_3_ = 14.662, *p* = 0.002), zona fasciculata (*H*_3_ = 21.139, *p* < 0.001), and zona glomerularis (*H*_3_ = 19.645, *p* < 0.001). Furthermore, in comparison with the HFHC group, the HFHC + TAA showed elevated CCO values in the zona fasciculata (*H*_3_ = 21.139, *p* < 0.001) and NC + TAA in the zona reticularis (*H*_3_ = 19.645, *p* < 0.001) (Figure 14).

## Discussion

**FIGURE 15:**
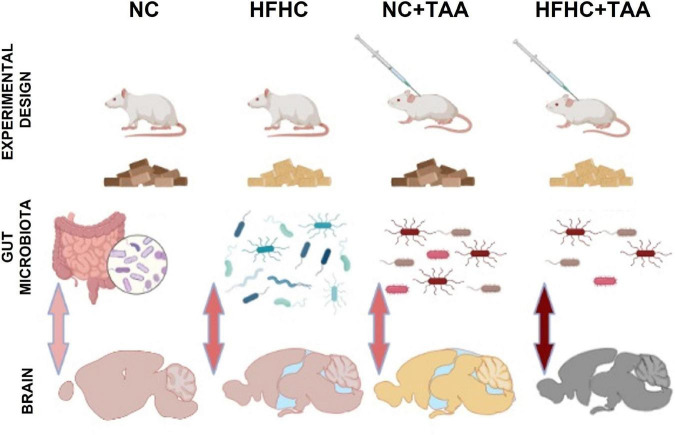
Graphic model of the study. The liver disease spectrum was studied in a rat model by using a high-fat, high-cholesterol diet (HFHC), control diet + thioacetamide (NC + TAA), and high-cholesterol diet + thioacetamide (HFHC + TAA). The experimental groups displayed changes in the gut microbiota at a compositional level, diversity, and bacterial community structure. Liver disease and gut dysbiosis also affected oxidative metabolism of several brain regions, which was associated with worsened object recognition and working memory.

This study aimed to investigate the evolution of the consequences of liver injury at different levels to disentangle the potential liver–gut–brain axis implication across the disease. For this purpose, we used an HFHC model, which emulates NASH, an NC + TAA model to simulate acute liver failure, and an HFHC + TAA model to assess the effect of the superimposed damages (Figure 15).

Our results showed that there were no statistically significant changes in body weight between groups across the weeks. However, regarding the relative organ weights, some differences were found, which were mainly in accordance with our previous study using HFHC administration (Higarza et al., 2019). These changes include a reduction in the relative weight of the brain displayed by the HFHC group compared with NC, and also the splenomegaly manifested by both groups fed with the HFHC diet in comparison with the NC group, which would indicate a strong effect of the nutrition values (Méndez-López et al., 2005). On its way, both groups injected with TAA displayed increased relative weight of the adrenal glands when compared with the NC group, which could be derived from an exacerbated stress response (Méndez-López et al., 2005). Finally, and as expected, as a typical feature of NASH (Xu et al., 2010), HFHC and HFHC + TAA groups show an increased liver weight when comparing both groups dispensed an NC diet, and so did HFHC when compared with HFHC + TAA. Besides, the liver microscopic study revealed steatosis in HFHC, fibrosis accumulation in NC + TAA, and combined hepatic damage in HFHC + TAA, which indicates an appropriate representation of human pathological processes in our models (Traussnigg et al., 2015; Schuppan et al., 2018).

The study of biochemical values in plasma revealed that the HFHC group displayed an elevated concentration of ammonia in comparison with the NC and NC + TAA groups, thus, this type of diet is able to provoke hyperammonemia (Higarza et al., 2019). Whereas unexpectedly this effect was not achieved by the isolated injection of TAA, the administration of both HFHC and TAA led to an exacerbated raise of ammonia levels, these being even higher than those presented by the HFHC group and representing the superimposed group, the more affected by hyperammonemia. The effect of the HFHC diet is also observed on cholesterol levels since the HFHC and HFHC + TAA groups displayed elevated values in comparison with NC and NC + TAA, which can be explained as a consequence of the elevated amount of cholesterol included in their diets. On its part, the HFHC diet also provoked, as expected, an elevation of ALT concentration (Higarza et al., 2019), since this value was increased in HFHC in comparison with NC + TAA and between HFHC + TAA and NC + TAA; however, when is not accompanied by the HFHC diet, TAA reduced ALT values in comparison with the NC group. Finally, both the TAA-injected groups displayed increased creatinine concentrations than the NC and HFHC groups, which is linked with the administration of this hepatotoxin (Shaikh Omar, 2018; Zargar et al., 2019) and is used as a biomarker of kidney function in patients with acute liver failure and cirrhosis (Piano et al., 2018; Hadem et al., 2019).

The study of the gut microbiota disclosed alterations on the composition and metabolism depending on the type of liver damage. In accordance with previous results (Higarza et al., 2019). NC animals showed higher relative abundances of *Lactobacillaceae* and *Ruminococacceae* than HFHC and in the HFHC group displayed elevated abundances of *Peptostreptococcaceae.* PCoA revealed differences among the studied groups, clearly marked by the diet employed for the emulation of steatosis and other typical features of the disease, a fact that was previously observed by our group (Higarza et al., 2019). However, pairwise comparisons also allowed to reveal significant dissimilarities between groups with and without thioacetamide on the same regimen of diet (HFHC vs. HFHC + TAA, and NC vs. NC + TAA), which means that the acute liver damage is affecting the bacterial community structure as well. Alpha diversity was also affected by the conditions, and a decrement in the Chao 1 index was observed both by diet (HFHC group; Higarza et al., 2019) and thioacetamide (NC + TAA, HFHC + TAA), with respect to the NC group. The superimposed group, HFHC + TAA, was the most affected, exhibiting significantly lower richness and evenness. It seems that the liver harm entailed a loss in rare species in all the groups, but less so in most abundant species, with the exception of the HFHC + TAA group with loss higher in both types of bacteria. Differences at the compositional level were also found among the experimental groups. Proteobacteria was found significantly higher in HFHC and HFHC + TAA groups, and the families *Burkholderiaceae* and *Enterobacteriaceae* were characteristics of those groups, respectively, when all the groups were compared among them. However, *Eubacterium fissicatena* group and *Lachnoclostridium*, genera that belonged to the Firmicutes phylum, were the discriminant taxons in the HFHC + TAA group when compared to the HFHC group. These results indicate that the effect of TAA on the microbiota is dependent on the basal microbiota, indeed, the impact of TAA is different in NC animals, that is, in animals in which microbial dysbiosis has already been introduced by the diet, i.e., HFHC group.

*Lactobacillus* was the dominant genus in the experimental groups with normal chow, an event that has been previously reported in the same animal strain (Higarza et al., 2019), and it was also the discriminant taxon when those groups were compared with the ones with a high-fat, high-cholesterol diet. However, the group with acute cirrhosis (NC + TAA) showed an elevated concentration of *Akkermansia* genus, characteristic and higher than in the other groups. Indeed, an acute liver disease condition could be linked to an attempt at the reinforcement of the gut barrier and reshaping of the perturbed gut microbiota by upregulating *Akkermansia* richness (Yang et al., 2020). Moreover, thioacetamide was responsible as well for an increment in the concentration of *Bifidobacterium*, which has been previously demonstrated to be negatively associated with total serum cholesterol (Zhang et al., 2021), independent of the regime of diet.

Another interesting finding is that no differences were found in *Lactobacillaceae* concentration between NC and NC + TAA, This is in line with previous studies which have demonstrated that Lactobacilli can efficiently treat hyperammonemia and hepatic encephalopathy by remodeling the intestinal microbiota and reducing the production and absorption of ammonia (Nicaise et al., 2008; Singh et al., 2018), as well as by downregulating the serum transaminase levels as seen in the ammonia and ALT levels of NC + TAA. The in-depth alterations in the gut microbiota shown by the different liver disease models across the spectrum have been described for the first time in this study.

Also, as expected from the differences observed in the composition of the intestinal microbiota, changes in the levels of the main SCFAs were observed but, in this case, the main differences were rendered by the diet groups, with lower concentrations on the HFHC and HFHC + TAA groups. However, in both groups treated with TAA, the levels of the BCFAs were lower than in their respective same diet group, which could support the results from Teck et al. (2004).

Furthermore, evidence exists in mice that acetate can alter the levels of glutamate, glutamine, and GABA (Frost et al., 2014), and some studies have shown that SCFAs and their metabolites can stimulate vagus nerve signaling (De Vadder et al., 2014; Li et al., 2018). Moreover, other studies have correlated changes in SCFAs and some BCFAs such as iso-butyrate with effects on behavior through the liver–gut–brain axis (Golubeva et al., 2017). Hence, these results could highlight the contribution of SCFAs and BCFAs to the cognitive dysfunction shown by HFHC, HFHC + TAA, and NC + TAA groups.

Regarding the behavioral evaluation, we found that there were no significant differences in motor activity between the groups, thus impaired execution in the task was not derived from a worsened locomotor function. To know the cognitive status of the models, we first assessed the recognition memory through the novel object and novel place tasks, which enables the examination of its “what” and “where” components. When we studied novel object recognition, we found that HFHC, NC + TAA, and HFHC + TAA displayed a worsened execution when discriminating the new object. On the other hand, in the novel place recognition test, whereas HFHC group showed a preference for the previous location of the object, which indicates a lack of innate preference for the novelty typically displayed by the animals (Berlyne, 1950), NC + TAA and HFHC + TAA were not capable of recognizing the change in place of the object, which to our knowledge has not been previously described. Deepening further into the memory processes, we evaluated spatial working memory, which is a type of short-term memory mainly dependent on the prefrontal cortex (Jeneson and Squire, 2012). Here, we could observe that the HFHC and HFHC + TAA groups were not able to remember the position of the platform across the days, whereas the NC + TAA group displayed a significant delay in the acquisition of the task.

Our studies are in line with the existing literature since the recognition of a novel object and spatial working memory have been previously shown to be altered as a consequence of HFHC diets (Higarza et al., 2019, 2021) and the administration of TAA (Méndez et al., 2008b; El-Marasy et al., 2019). In humans, although this field has not been widely addressed, some studies have described that patients with NAFLD/NASH spectrum displayed certain cognitive impairment, including visuospatial and frontal-associated deficits (Celikbilek et al., 2018; Moretti et al., 2019). Likewise, acute and also chronic liver damage have been linked to neuropsychiatric symptoms encompassed in hepatic encephalopathy dysfunction, including worsened attention and memory (Weissenborn et al., 2005; Weissenborn, 2019). In this way, liver damage is linked with cognitive impairment from the early stages to the more advanced phases.

To disentangle the mechanisms underlying these cognitive impairments, we studied brain oxidative metabolism through the CCO technique. Here, we could observe that the superimposed model was the most affected group since it displayed region-specific elevated oxidative metabolism in comparison with the rest of the groups, indicating an abnormal activation during the execution of the task. In this way, HFHC + TAA showed higher CCO values in the prefrontal, retrosplenial, and perirhinal cortices in comparison with the HFHC group, in the amygdala compared with NC and HFHC groups, and in the mammillary nuclei compared with the HFHC and NC + TAA groups.

These results point out that, although the cognitive deterioration in this group was as severe as that manifested by the groups representing NASH and acute liver damage, the superimposed model demanded an increased oxidative activity in some specific brain regions to attempt to accomplish the spatial working memory task. These results are similar to what has been observed in TAA-induced cirrhosis (Méndez et al., 2009), indicating therefore an enhanced brain dysfunction resulting from the combination of the different hits.

The prefrontal cortex is classically associated with working memory (Jeneson and Squire, 2012), but it requires the support of a complex network as it is known that it is in close relation with retrosplenial and perirhinal cortices, which not only sustain working memory but also object recognition (Apergis-Schoute et al., 2006; Mendez et al., 2015; Bubb et al., 2017; Powell et al., 2017), and even with diencephalic areas such as the mammillary bodies (Conejo et al., 2004). The amygdala is also in connection with the prefrontal cortex and is implicated in stress response (McEwen et al., 2016), being more activated in the HFHC + TAA group in comparison with the non-TAA-administered groups. This result could be in line with the results derived from the study of the adrenal glands. Indeed, it is known that adrenal stress hormones can modulate memory consolidation through an interaction with the amygdala, which, in turn, displays efferent projections with other brain regions (Roozendaal et al., 2007).

Our results showed that, when compared with the NC group, NC + TAA, and HFHC + TAA groups showed not only an elevated relative adrenal weight but also higher CCO values in the zona glomerulosa, the fasciculata, and the reticularis, which secrete mineralocorticoids, glucocorticoids, and adrenal androgens, respectively (Mitani, 2014). Moreover, even when compared with the HFHC group, the HFHC + TAA showed elevated CCO values in the zona fasciculata and NC + TAA in the zona reticularis. Therefore, these models of advanced liver damage could implicate the hypothalamic–pituitary–adrenal axis as a stress response (Méndez-López et al., 2005).

These mitochondrial alterations could be a consequence of the hepatic disease since it is known that cirrhosis has been associated with elevated activity of CCO (Krähenbühl and Reichen, 1992), and liver damage can provoke oxidative stress since it is linked with the production of free radicals that influence the balance between free radicals and antioxidative mechanisms (Méndez et al., 2009; Cichoz-Lach and Michalak, 2014). Indeed, hepatic encephalopathy is known to be associated with oxidative stress and mitochondrial dysfunction (Norenberg et al., 2004; Dhanda et al., 2018; Heidari, 2019), and the brain of the patients affected by this disorder is particularly sensitive to this type of damage (Norenberg et al., 2004). Besides, it has been described that oxidative stress can be given as a consequence of elevated levels of ammonia (Maltez et al., 2017; Wang et al., 2018; Zhang et al., 2018; Heidari, 2019), which has been also associated with increased brain oxidative metabolism demands (Arias et al., 2014) and is known to contribute to the cognitive impairment in hepatic encephalopathy (Felipo et al., 2012). Henceforth, progressive liver damage would be leading to several pathways, which would result in brain and adrenal mitochondrial dysfunction, being reflected in impaired cognitive function.

This study is a pioneer in highlighting the dynamic changes of the gut microbiota and cognition in the progression of liver disease. The systemic results along the liver–gut–brain axis shed light on the opportunity to manipulate the gut microbiota and its related metabolites as effective strategies in preventing brain-associated liver disease dysfunction.

## Data availability statement

The original contributions presented in this study are included in the article/Supplementary material, further inquiries can be directed to the corresponding author.

## Ethics statement

The animal study was reviewed and approved by University of Oviedo.

## Author contributions

JA, MG, and NA contributed to the conception and design of the study. SH performed the experiments. SH and SA analyzed the samples. SH, SA, and NA carried out the statistical analysis and wrote the manuscript. All authors contributed to the manuscript revision, read, and approved the submitted version.
