# Supplementary material for: The gut–microbiota–brain changes across the liver disease spectrum
Source: Front Cell Neurosci. 2022 Sep 7;16:994404. doi: 10.3389/fncel.2022.994404 (PMC9490445; doi:10.3389/fncel.2022.994404)
Supplement: Supplementary file 1 [file Presentation_1.PPTX]

## Slide 1
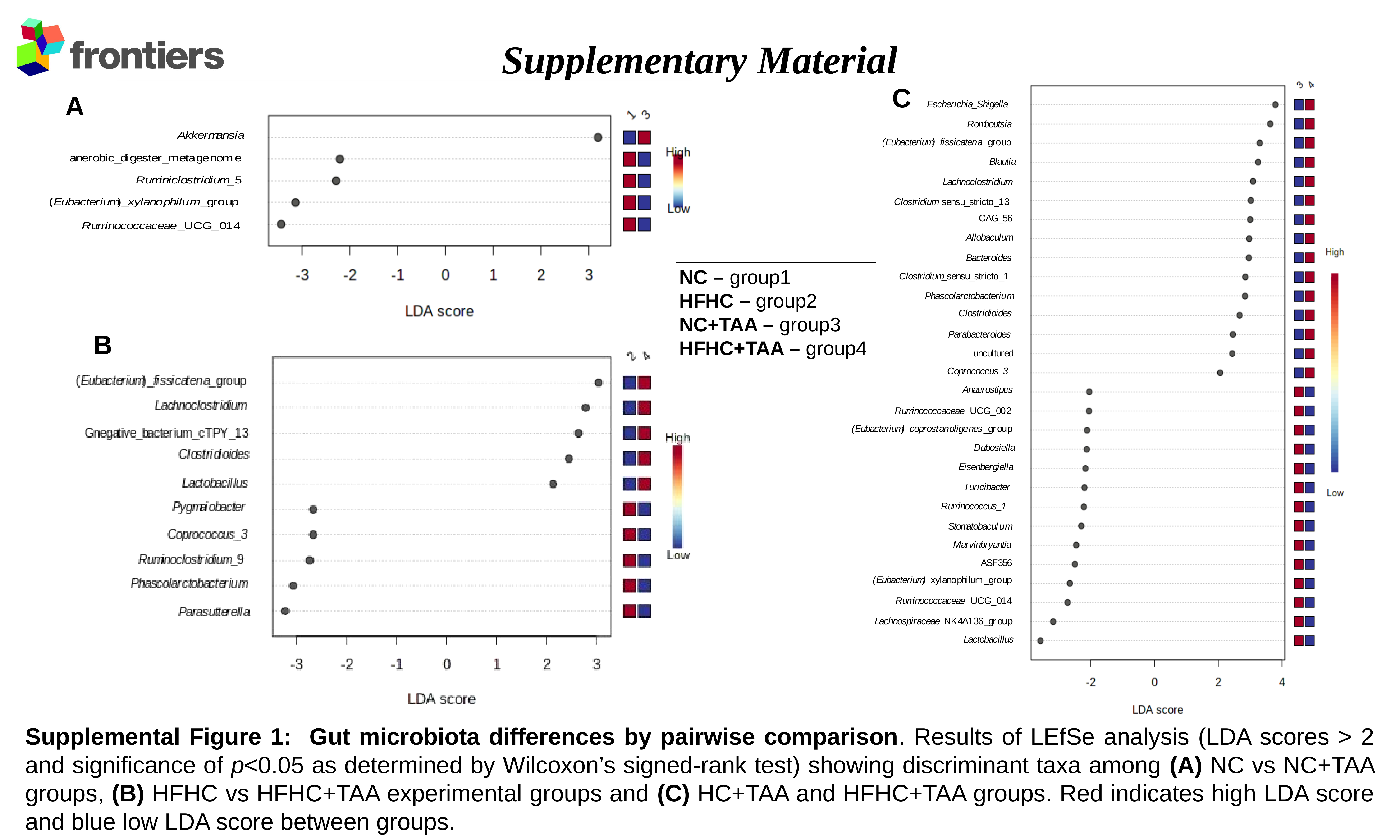

Supplementary Material
C
A
NC – group1
HFHC – group2
NC+TAA – group3
HFHC+TAA – group4
B
Supplemental Figure 1: Gut microbiota differences by pairwise comparison. Results of LEfSe analysis (LDA scores > 2 and significance of p<0.05 as determined by Wilcoxon’s signed-rank test) showing discriminant taxa among (A) NC vs NC+TAA groups, (B) HFHC vs HFHC+TAA experimental groups and (C) HC+TAA and HFHC+TAA groups. Red indicates high LDA score and blue low LDA score between groups.
